# Selective Retina Therapy with Real-Time Feedback-Controlled Dosimetry for Treating Acute Idiopathic Central Serous Chorioretinopathy in Korean Patients

**DOI:** 10.1155/2018/6027871

**Published:** 2018-02-06

**Authors:** Ye Ji Kim, Youn Gon Lee, Dong Won Lee, Jae Hui Kim

**Affiliations:** Department of Ophthalmology, Kim's Eye Hospital, Konyang University College of Medicine, Seoul, Republic of Korea

## Abstract

**Purpose:**

To evaluate short-term treatment outcomes following selective retina therapy (SRT) with real-time feedback-controlled dosimetry in Korean patients with acute idiopathic central serous chorioretinopathy (CSC).

**Methods:**

Sixteen eyes (16 patients) with acute idiopathic CSC (symptom duration < 3 months) were included in this retrospective study. All patients underwent a single session of SRT with real-time feedback-controlled dosimetry. Best-corrected visual acuity (BCVA) and central foveal thickness (CFT) before and 3 months after treatment were examined and compared.

**Results:**

The logarithm of minimal angle of resolution BCVA was significantly better 3 months after treatment (0.16 ± 0.18) than at the time of diagnosis (0.27 ± 0.18, *P* = 0.002). Additionally, subretinal fluid had resolved in all 16 eyes 3 months after treatment and CFT was significantly lower 3 months after treatment (215.6 ± 17.9 *μ*m) than at baseline (441.4 ± 124.8 *μ*m, *P* < 0.001). No notable SRT-related complications were observed during the study period.

**Conclusion:**

The results of the present study suggest that SRT is a useful therapeutic option for patients with acute idiopathic CSC. Further studies are required to better understand the long-term efficacy of this treatment. This trial is registered with clinical trial registration number NCT03339856.

## 1. Introduction

Central serous chorioretinopathy (CSC) is a disorder that is characterized by a localized serous detachment of the neurosensory retina in the posterior pole [1]. Additionally, CSC is often the self-limiting and spontaneous resolution of subretinal fluid (SRF) which often occurs within several months [2]. Although several treatment modalities (e.g., laser photocoagulation, [3] photodynamic therapy [4], and antivascular endothelial growth factor (VEGF) therapy [5]) have been used in CSC patients, a consensus has not yet been reached on the optimal time for intervention.

Selective retina therapy (SRT) selectively disrupts the retinal pigment epithelium (RPE) with minimal damage to the photoreceptors [6]. Previous studies have shown SRT to be effective for resolving SRF, while causing only minimal collateral damage to the retina and vision [7–12]. However, most patients included in prior studies had chronic CSC (≥3 months symptom duration) and SRT efficacy on acute CSC is not fully known.

When treating CSC patients, it is generally recommended to follow up several months without any intervention to identify spontaneous resolution of SRF. However, prompt treatment may be beneficial for some patients. For example, metamorphopsia or micropsia associated with CSC can greatly interfere with driving or with work when an occupation requires delicate procedures. Additionally, patients are often psychologically distressed by a CSC diagnosis and the accompanying decrease in the vision-related quality of life [13, 14]. In these cases, prompt treatment to reduce SRF may relieve, at least in part, CSC symptoms and their related stress.

The current study evaluated short-term treatment outcomes following SRT with real-time feedback-controlled dosimetry in Korean patients with acute idiopathic CSC.

## 2. Materials and Methods

This retrospective study was performed at a single center. All study conducted adhered to the tenets of the Declaration of Helsinki. This study was approved by the Kim's Eye Hospital institutional review board.

### 2.1. Patients

This study included patients who were diagnosed with treatment-naïve idiopathic CSC and were treated with SRT. The R:GEN device (Lutronic, Goyang-si, South Korea) was used to administer SRT, as was done in a previous study by Park et al. [10]. Inclusion criteria also included SRF involving the fovea (documented by optical coherence tomography (OCT) performed at initial presentation) and symptom duration shorter than 3 months. Patients were excluded from analyses if any of the following were true: (1) age > 55 years; (2) clinical or angiographic features suggestive of choroidal neovascularization; (3) OCT findings suggestive of type 1 neovascularization or polypoidal choroidal vasculopathy (e.g., double layer sign or fibrovascular pigment epithelial detachment); (4) history of macular laser photocoagulation, photodynamic therapy, or anti-VEGF therapy; or (5) history of exogenous corticosteroid treatment for a systemic disease (e.g., Cushing's syndrome or renal disease).

### 2.2. SRT Device

The R:GEN device uses a Q-switched Nd:YLF laser with a wavelength of 527 nm, a pulse duration of 1.7 *μ*s, and a repetition rate of 100 Hz. A single-mode Gaussian laser beam is delivered from the cavity to the slit lamp by a multimode optical fiber with a 50 *μ*m core diameter. The slit lamp has parfocal zoom optics that deliver a 200 *μ*m diameter beam to the human retina. The SRT laser system with a real-time dosimetry feedback system has received CE marking from an EU Notified Body (approved on December 21, 2015) and has been approved by the MFDS (Ministry of Food and Drug Safety in Korea) for CSC and diabetic macular edema (approved on March 31, 2015).

**Figure 1:**
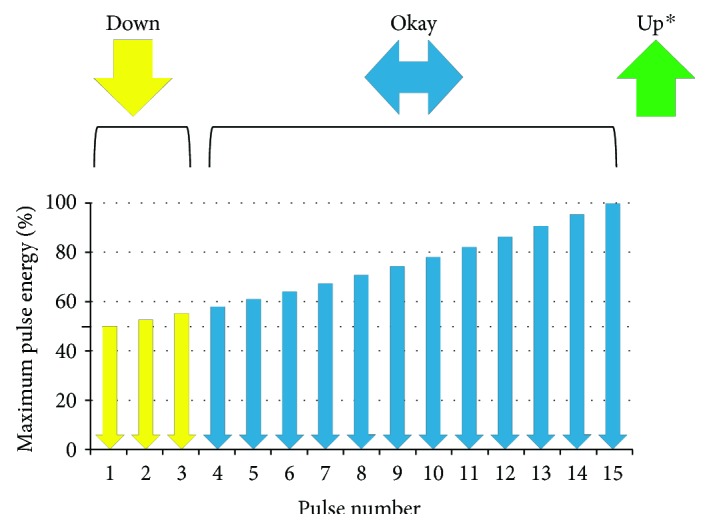
The laser pulse energy was emitted in a stepwise fashion (an increment of 3.57% for the following micropulse) for every individual spot. When there is an adequate feedback signal from the target retinal pigment epithelial (RPE) cells, the device stops the laser automatically during the 15-pulse set of a shot. If a shot was stopped at or between pulse numbers 4 and 15, the energy level would be deemed adequate for the shot and also adequate for the next shot. If the shot was stopped at earlier pulses, between pulse numbers 1 and 3, the shot's treatment was adequate for that spot by having received desired feedback from RPE, but for the next shot, the device recommends a decreased energy level to ensure the safety of the next shot. Conversely, if the desired feedback from a set of 15 pulses is not achieved, that is, no bubble is detected, then the energy level settings are too low. In this case, the device recommends incremental energy increases to achieve the desired feedback from RPE. ^∗^Up only if the laser was not stopped in 15 pulses.

The laser system delivers 15 consecutive micropulses in a burst of stepwise energy-ramping pulse trains. The last (15th) and maximum energy pulse can be adjusted by the physician for each treatment spot as a preset value. The first energy pulse is 50% of the preset value, and the energy of the following micropulse gradually increases by an additional 3.57% to reach 100% at the 15th pulse (Figure 1). As the energy pulse of the laser is raised, the temperature of the melanosomes in the RPE cells within the laser-irradiated area increases, and microbubbles are generated. This microbubble generation can be monitored with ultrasound and reflectometric sensors [10, 15, 16].

For acoustic signal measurement, a ring-shape piezoceramic sensor is embedded in a contact lens placed on the patient's eye by the physician. The detected signal is amplified, and then the signal is processed in several steps, including offset removal, rectification, and summation. The final processing outcome is termed an optoacoustic value (OAV), which is an arbitrary value. The implemented optoacoustic (OA) algorithm employs the OA signal from microbubble formation to detect the rupture of the RPE cell membrane using a predetermined threshold. The reflectometric (RM) signal was also obtained using an avalanche photodetector (APD120A2/M, THORLABS, Newton, USA) located in the middle of the optical path. The photodetector measures the scattered laser signal from the microbubbles generated in the RPE cells. The detected optical signal is processed during the rectification, summation, noise subtraction, and normalization filtering steps. There are more processing steps for the RM signal compared with the OA because it is necessary to remove the influence of energy ramping. The final outcome of this process is termed the reflectometric value (RMV), which is also an arbitrary value. Like the OAV, the RMV is considered to indicate RPE cell damage as soon as it exceeds a predetermined threshold value. The OA and RM are related to the “OR” logic in the dosimetry algorithm. Therefore, the feedback of the OA and RM signals from microbubble formation indicates whether there is RPE cellular damage when either of the feedback signals is above the threshold after each individual laser micropulse is delivered. The threshold value was set as 2.0 for OA and 6.0 for RM. As soon as the threshold is exceeded by the detection of microbubble formation, the subsequent burst of micropulses is automatically stopped.

The fundamental real-time feedback-controlled dosimetry (RFD) algorithm of the automatic laser shutoff used in our study was similar to that used in the study by Park et al. [10] One difference was that the “up arrow sign,” which signaled the operator to increase laser energy, is presented when a shot was stopped at the 13th, 14th, and 15th pulse in the previous study [10], whereas the “up arrow sign” was not presented in the same situation by the device used in our study. The “up arrow sign” was presented only when microbubble formation was not detected even after all 15 consecutive micropulses. This modification was performed to avoid using unnecessarily high laser energy.

### 2.3. Examination, Treatment, and Follow-Up

All subjects underwent comprehensive ophthalmologic examinations, including best-corrected visual acuity (BCVA) measurement, slit-lamp biomicroscopy (90-diopter lens), fluorescein angiography (FAG), fundus photography, and spectral domain OCT (Spectralis HRA-OCT™, Heidelberg Engineering, Dossenheim, Germany, or RS 3000™, Nidek Co. Ltd., Tokyo, Japan). Indocyanine green angiography using a confocal laser-scanning system (Spectralis HRA-OCT) was performed at the discretion of each physician. A CSC diagnosis was based on FAG findings, in particular, evidence of a typical ink blot or smokestack leakage. A single examiner (J.H.K.) reviewed all FAG results.

All SRT were performed by a single physician (J.H.K.) using a Nd:YLF laser (wavelength of 527 nm, 15 micropulses per spot, single micropulse duration of 1.7 *μ*s, and pulse repetition rate of 100 Hz). The spot size was set at a diameter of 200 *μ*m. The RFD system [10, 15, 16] was used to determine laser energy for treatment. Before laser application to the leakage points, test shots were performed to determine laser energy required for treating the leakage points. The laser energy for test shots began at 80 *μ*J and was increased in 5–10 *μ*J intervals until an okay sign, which suggested intracellular bubble formation, was indicated on the RFD system panel. In general, several laser shots were required to achieve an okay sign. Following this confirmation step, the same laser energy when the okay sign was showed was applied to fluorescein leakages. The purpose of the test shots was to avoid unnecessary damage to the macular area during the titration of laser energy. For this reason, test shots were applied outside of the superior or inferior temporal arcade.

**Figure 2:**
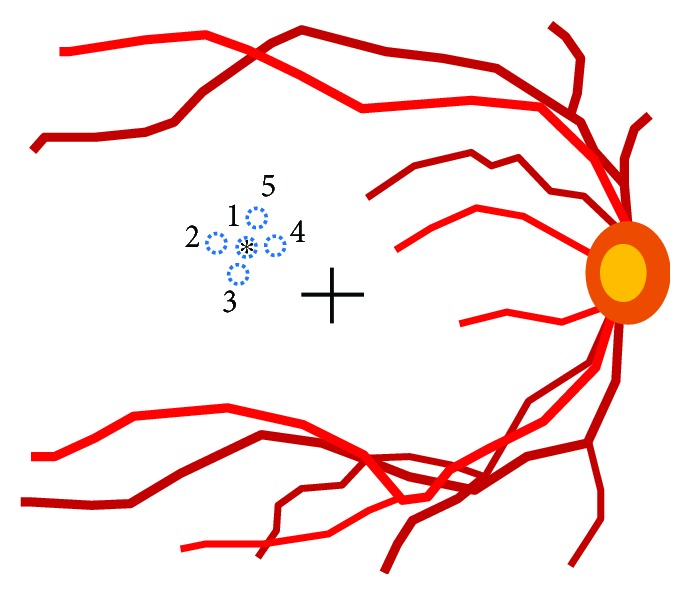
A depiction showing the location of laser spots. Initially, a laser spot was applied to the leakage point (asterisk). Next, several additional laser spots were applied around the leakage point. The distance between the spots was generally set as 0.5 to 1.0 spot diameter (100 to 200 *μ*m). Blue dotted circles indicate the location of laser spots. The numbers (1 to 5) indicate the order of laser spots.

If the device indicated an okay sign at leakage points, laser shots using the same energy were administered around the leakages (Figure 2). If the okay sign was not indicated at the leakage points, laser energy would again be increased in 5–10 *μ*J increments until the appearance of the okay sign. If the device control panel indicated that excessive laser energy had been applied (down arrow sign), the laser energy was decreased in 5–10 *μ*J increments until the okay sign was again indicated. The total number of laser shots delivered around fluorescein leakage points that were at or above the laser energy when the okay sign was showed was counted.

Patients had follow-up visits at 1 and 3 months after SRT, at which BCVA was measured and OCT examination was performed. Central foveal thickness (CFT) was manually measured on OCT images and was defined as the distance between the RPE and the internal limiting membrane.

### 2.4. Outcome Measures

Both BCVA and CFT were measured at diagnosis, at 1 and 3 months following SRT, and at the final follow-up visit. The incidence of complete SRF resolution was estimated. Color fundus photographs taken at each follow-up visit also documented laser treatment and were used to check for the presence of visible laser spots. All BCVA measurements were converted to the logarithm of the minimum angle of resolution (logMAR) for data analyses. The minimal and maximal energy was defined as the minimal and maximal laser energy used for laser shots applied to or around the leakage points, respectively. For patients who were followed up more than three months later, the entire follow-up data was analyzed to verify any recurrence of fluid. The information regarding these patients was presented as additional data.

### 2.5. Statistical Analyses

Statistical analyses were performed using a commercially available software package (SPSS v. 12.0 for Windows; SPSS Inc., Chicago, IL). Comparisons between BCVA and CFT measurements at different time points were performed using Wilcoxon signed-rank tests with a Bonferroni correction. Statistical significance was defined as *P* < 0.05.

## 3. Results

**Figure 3:**
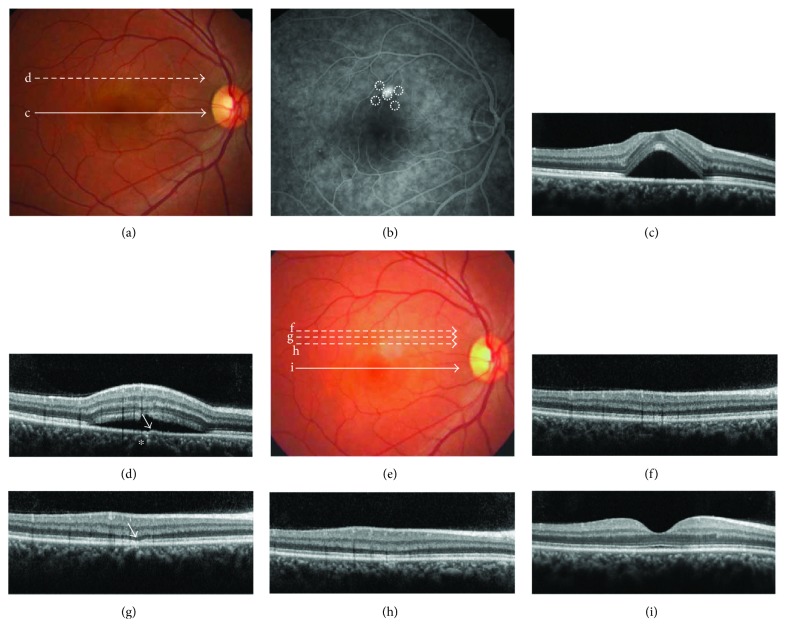
Fundus photographs (a, e) as well as fluorescein angiographic (b) and optical coherence tomographic (OCT) (c, d, f–i) images of a 38-year-old patient with acute central serous chorioretinopathy treated with selective retina therapy (case number 5 in Tables 1 and 2). At diagnosis (a–d), best-corrected visual acuity (BCVA) was 20/25, and the patient complained of metamorphopsia. At the location of leakage, a shallow pigment epithelial detachment (PED) (d) (arrow) was observed adjacent to the pachy vessel (d) (asterisk). Three months after treatment (e–i), subretinal fluid had completely resolved and BCVA had improved to 20/20. A PED had resolved after treatment with thinning of the ellipsoid zone at the location of previous leakage (panel g, arrow). There was no notable treatment-related retinal pigment epithelial damage (f-g). The dotted circles in (b) indicate laser spots. Lowercase letters in panels (a) and (e) indicate OCT scanning lines for panels with the same letters.

Sixteen eyes of 16 patients (13 men and 3 women) were included in this study. Mean patient age was 44.6 ± 7.3 years. All patients had treatment-naïve CSC, and all patients were treated with a single SRT session.  Figure 3 shows treatment outcomes of a representative case.

**Figure 4:**
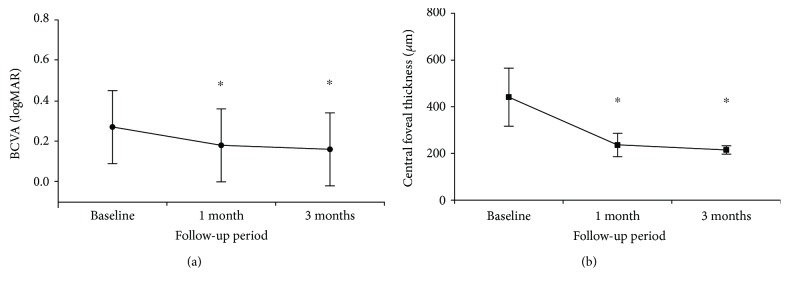
Changes in best-corrected visual acuity (BCVA) (a) and central foveal thickness (b) after selective retinal therapy. There was a significant improvement in BCVA accompanied with significant decrease in central foveal thickness after treatment. Asterisks indicate significant difference when compared to the baseline value. logMAR: logarithm of minimal angle of resolution.

Three eyes exhibited two leakage points. In the remaining 13 eyes, only one leakage point was noted. The mean distance between the fovea and the leakage points (19 points from 16 eyes) was 1119.2 ± 525.3 *μ*m. There was no subfoveal leakage point, and the juxtafoveal leakage point (≤200 *μ*m) was noted in one eye. The leakage points were extrafoveal in the remaining 15 eyes. Patients were treated with a mean of 5.4 ± 1.2 laser shots (not including test shots, range: 4 to 8 shots) around leakage points. The mean maximal laser energy was 105.0 ± 14.6 *μ*J (range: 80 to 140 *μ*J). Characteristics of and treatment outcomes for each patient are summarized in Tables 1 and 2. Mean BCVA at diagnosis, 1 month, and 3 months after SRT was 0.27 ± 0.18 (Snellen equivalent: 20/81), 0.18 ± 0.18 (20/55), and 0.16 ± 0.18 (20/57), respectively (Figure 4(a)). The BCVA at 1 month (*P* = 0.002) and 3 months (*P* = 0.002) following SRT were both significantly better than that at baseline. Additionally, at 3 months, BCVA had improved from baseline in 14 of 16 patients (87.5%) and had been maintained in the remaining 2 patients (12.5%).

All eyes had SRF at baseline, and 5 eyes (31.3%) had residual SRF at 1 month. However, at 3 months, SRF had resolved in all 16 patients. Additionally, SRF did not increase or recur in any patient during the 3-month follow-up period. Quantitative CFT measurements were in agreement with clinical observations. The CFT was 441.4 ± 124.8 *μ*m, 236.5 ± 50.4 *μ*m, and 215.6 ± 17.9 *μ*m at baseline, 1 month, and 3 months, respectively (Figure 4(b)). Mean CFT at 1 and 3 months was significantly different than at baseline (both *P* < 0.001). Additionally, all 16 patients had a reduction in CFT by 3 months.

No SRT-related complications were observed. Laser spots were not detectable on color fundus photographs, and no patient complained of new or worsening symptoms.

Among the 16 patients, eight patients were followed up at five to seven months after treatment (Table 2). Among the eight patients, recurrence of SRF was not noted during OCT examination in five patients. In the remaining three patients, recurrence of SRF was not noted during clinical fundus examination.

## 4. Discussion

In our study of acute idiopathic CSC treatment, improvements of both SRF resolution and visual acuity were noted after SRT. Three months after SRT, all 16 patients included in this study had complete SRF resolution. Additionally, visual acuity improved in 14 of 16 patients (87.5%) and was maintained in the remaining 2 patients (12.5%). Furthermore, no notable SRT-related complications were observed.

Central serous chorioretinopathy is often self-limiting. Therefore, treatment safety is as important as treatment efficacy for cases of acute CSC. Widely used treatments for CSC include conventional laser photocoagulation, photodynamic therapy, and intravitreal anti-VEGF therapy. Although conventional laser photocoagulation effectively resolves SRF, thermal damage to adjacent retinal tissue inevitably occurs [17, 18]. As a result, a scotoma or choroidal neovascularization can develop in some patients [19]. Photodynamic therapy is another effective treatment that causes less damage to adjacent tissue than laser photocoagulation does [20–22]. However, photodynamic therapy has some drawbacks, including its high cost and the systemic side effects associated with intravenous verteporfin use. Photodynamic therapy is also inconvenient for patients because they must maintain a dark environment after treatment to avoid phototoxicity. Anti-VEGF therapy is a simple and safe treatment for CSC [5, 23, 24]. However, there has been controversy regarding anti-VEGF therapy efficacy in the eyes with CSC, [25, 26] especially in younger patients [24]. Subthreshold laser therapy [27, 28] and mineralocorticoid antagonist [29, 30] are other treatment options for patients with CSC. Although both therapies are effective, more evidence is required to firmly establish the pros and cons of these treatments. Because the gold standard treatment for CSC has not yet been agreed upon, broadening treatment options for this condition would be valuable.

Selective retinal therapy was developed to target retinal diseases likely associated with RPE degradation [31]. The RPE is the target tissue of SRT, and the overlying retina is only minimally impacted. This is because the short SRT laser pulses allow energy to be confined to the RPE, which minimizes heat conduction to adjacent tissue [31, 32]. Using the minimally destructive laser energy allows for effective treatment of a selected lesion while minimizing unnecessary cell damage. Real-time optoacoustic dosimetry was developed to help determine what minimally destructive laser energy should be [16]. During SRT, laser energy is mainly absorbed by the intracellular melanosomes of RPE cells, which results in microbubble formation [33, 34]. Schuele et al. [16] demonstrated that microbubble formation around melanosomes can be detected using optoacoustic dosimetry. The current study used an automatic real-time feedback-controlled dosimetry system mounted on the R:GEN device. A previous study on patients with chronic CSC found this system to be effective for determining the laser energy required for treatment [10].

An early clinical study examined SRT for treating patients with diabetic maculopathy, soft drusen, and CSC [12]. Following treatment, Roider et al. [12] observed resolution of hard exudates and leakage in eyes with diabetic maculopathy, of drusen in eyes with soft drusen, and of SRF in eyes with CSC. Following this initial study, various investigations found that SRT is also effective for treating CSC [7–11]. Eyes with chronic CSC had an improvement in visual acuity following treatment with an accompanying decrease in SRF [7–11]. Additionally, treated patients were not left with a permanent scotoma [11]. To date, only 19 patients (over 2 studies) have been examined to determine outcomes following SRT performed to treat acute CSC (symptom duration ≤ 3 months) [7, 8]. Elsner et al. [7] found that 8 of 9 patients with acute CSC had complete resolution of SRF following treatment. Visual acuity improvements were noted in 8 of 9 patients. The remaining patient had a visual acuity of 20/20 before treatment, which was maintained 4 weeks after treatment. Framme et al. [8] observed complete SRF resolution in 10 of 10 patients examined. Visual acuity improvements were noted in 9 patients. However, 1 patient developed a small hemorrhage at the laser site, which completely resolved by 3 months.

The two previous studies that examined the effect of SRT on acute CSC did not mention patient ethnicity [7, 8]. However, both studies were conducted in Europe (Germany and Switzerland), making it likely that the majority of included patients were Caucasian. Because CSC incidence is higher in Asians than in Caucasians [35, 36], CSC treatments are especially important for Asian populations. The current study is the first to report treatment outcomes for SRT with real-time feedback-controlled dosimetry performed in Asian patients to treat acute idiopathic CSC. Our results are comparable to those of previous studies conducted in European countries [7, 8]. This observation suggests that SRT offers similar efficacy for treating acute idiopathic CSC, regardless of ethnicity.

Previous studies have used high-resolution OCT to examine eyes with CSC and found focal RPE defects [37–39]. Gupta et al. [38] postulated that, in the eyes with CSC, an increase in choroidal pressure can cause a pigment epithelial detachment and that further mechanical stress can cause RPE defects within the pigment epithelial detachment. Furthermore, these RPE defects ultimately lead to the development of a serous detachment. These defects often resolve following laser photocoagulation [37] or spontaneous SRF resolution [38]. In patients included in the current study, we assume that RPE remodeling after SRT led to a partial or complete closure of RPE defects and a subsequent decrease in SRF. Unfortunately, we did not perform dense serial OCT scans that covered fluorescein leakage points. Therefore, it was not possible for us to identify RPE defects. However, laser spot size was relatively large (diameter of 200 *μ*m) and multiple shots were applied around leakages. Given that RPE defect location usually coincides with fluorescein leakage location [37], SRT laser energy was most likely delivered to RPE defect sites. Proving this theory is beyond the scope of the present study, and further studies investigating changes in RPE layer morphology before and after SRT are needed.

In previous studies with SRT, the range of laser energy used to treat CSC was from 116.3 *μ*J to 168.6 *μ*J [8–10]. The mean maximal laser energy used in the present study (mean 105.0 *μ*J) was slightly lower than those used in previous studies. We postulate that the characteristics of laser machines used in the studies may be responsible for this difference. In fact, the mean laser energy used in the present study was similar to that used in the study by Park et al. (116.3 *μ*J), which used the same machine with a real-time dosimetry feedback system as that used in the present study.

The number of laser shots used in the present study was markedly lower than those used in previous studies with other types of nondamaging retinal lasers [40]. We posit that the primary reason for this difference is the method of counting shots used in our study. The laser shot was counted as “one” when an okay sign or down arrow sign was noted on the control panel. If the laser energy was not sufficient to achieve an okay sign, this shot was not counted. In addition, the number of shots did not reflect the total number of micropulses. In previous studies with other types of nondamaging retinal lasers, a single micropulse was counted as “one shot” [40]. In the present study, however, we did not count the number of total micropulses. As described in Materials and Methods, the laser system delivers up to 15 consecutive micropulses in a burst of stepwise energy-ramping pulse trains. Unfortunately, the number of micropulses delivered was not recorded in the electronic medical record system. The number of laser shots was counted as “one” even when all 15 consecutive micropulses were delivered.

The current study had several limitations. First, this was a retrospective study with a short follow-up period. In addition, there was no control group. Second, tests to evaluate retinal function (e.g., multifocal electroretinography, and visual field testing) were not performed. In addition, autofluorescence images or fluorescein angiographic images, which may detect RPE damage, were not taken after treatment. Therefore, we could not accurately evaluate whether or not there was treatment-associated damage to the retina. There was no established method to determine the number of laser spots, and the number of spots was arbitrarily determined at the discretion of the treating physician on a case-by-case basis. Since most of the leakage points were extrafoveal, the efficacy of SRT for subfoveal or juxtafoveal leakages requires confirmation through further study. Lastly, study patients were evaluated with one of two different OCT devices. Therefore, variations in retinal thickness measurements may have influenced our CFT results.

In summary, we evaluated the efficacy of SRT for treating acute CSC in Korean patients. In all the included patients, complete resolution of SRF accompanied with an improvement in visual acuity was noted 3 months after SRT. Our results suggest that SRT may be a useful therapeutic option for patients with acute idiopathic CSC. Further studies are required to determine long-term treatment efficacy in the eyes with acute CSC. In addition, we hope further studies with more frequent OCT examinations may reveal the exact time of SRF resolution after SRT.

## Figures and Tables

**Table 1:** Baseline characteristics and treatment for 16 included patients.

Case number	Age (years)	Sex	Number of leakage points	Location of leakage points	Number of laser shots	Energy, *μ*J	
						Minimum	Maximum
1	54	F	2	Extrafoveal	7	120	140
2	40	M	1	Extrafoveal	4	90	90
3	48	M	1	Extrafoveal	5	100	110
4	34	M	1	Extrafoveal	6	80	90
5	38	M	1	Extrafoveal	5	105	120
6	35	M	2	Extrafoveal	7	100	100
7	45	M	1	Juxtafoveal	5	90	100
8	35	M	1	Extrafoveal	4	95	100
9	39	M	2	Extrafoveal	8	100	110
10	50	M	1	Extrafoveal	4	80	80
11	48	M	1	Extrafoveal	5	100	100
12	51	M	2	Extrafoveal	5	95	110
13	53	F	1	Extrafoveal	5	105	110
14	53	F	1	Extrafoveal	5	100	100
15	39	M	1	Extrafoveal	7	95	120
16	52	M	1	Extrafoveal	5	90	90

F: female; M: male.

**Table 2:** Treatment outcomes for 16 included patients.

Case number	Best-corrected visual acuity			Central foveal thickness (*μ*m)			Subretinal fluid			Clinical course after 3 months
	Baseline	1 M	3 M	Baseline	1 M	3 M	Baseline	1 M	3 M	
1	20/60	20/60	20/50	507	314	197	+	+	−	No recurrence of fluid for 7 M confirmed by clinical examination
2	20/30	20/25	20/20	483	267	223	+	+	−	No recurrence of fluid for 6 M confirmed by OCT
3	20/50	20/40	20/30	657	388	252	+	+	−	No recurrence of fluid for 6 M confirmed by clinical examination
4	20/25	20/20	20/20	528	241	223	+	+	−	
5	20/25	20/20	20/20	488	228	226	+	−	−	No recurrence of fluid for 5 M confirmed by clinical examination
6	20/50	20/40	20/40	621	223	226	+	−	−	No recurrence of fluid for 5 M by OCT
7	20/40	20/25	20/20	316	222	228	+	−	−	No recurrence of fluid for 6 M by OCT
8	20/25	20/20	20/20	320	212	216	+	−	−	
9	20/20	20/20	20/20	499	229	223	+	−	−	
10	20/30	20/25	20/25	584	234	231	+	−	−	No recurrence of fluid for 6 M by OCT
11	20/25	20/20	20/20	444	213	206	+	−	−	
12	20/60	20/50	20/50	284	188	187	+	−	−	No recurrence of fluid for 5 M by OCT
13	20/50	20/40	20/40	290	211	204	+	+	−	
14	20/30	20/30	20/30	341	220	223	+	−	−	
15	20/30	20/25	20/25	422	203	201	+	−	−	
16	20/40	20/30	20/30	278	191	183	+	−	−	

M: month; +: present; −: absent; OCT: optical coherence tomography.

## Supplementary Materials

Supplementary Material Photographs of the R:GEN selective laser therapy device (Lutronic, Goyang-si, South Korea) used in this study (A). The system includes a combined contact lens/ultrasonic transducer for performing optoacoustic measurements (B). The real-time feedback system control panel displays an up arrow to indicate insufficient laser power. A horizontal double-headed arrow is the okay sign, which indicates microbubble formation (C).
