# Supplementary figures and images for: Selective Retina Therapy with Real-Time Feedback-Controlled Dosimetry for Treating Acute Idiopathic Central Serous Chorioretinopathy in Korean Patients
Source: J Ophthalmol. 2018 Feb 6;2018:6027871. doi: 10.1155/2018/6027871 (PMC5818953; doi:10.1155/2018/6027871)

## Slide 1
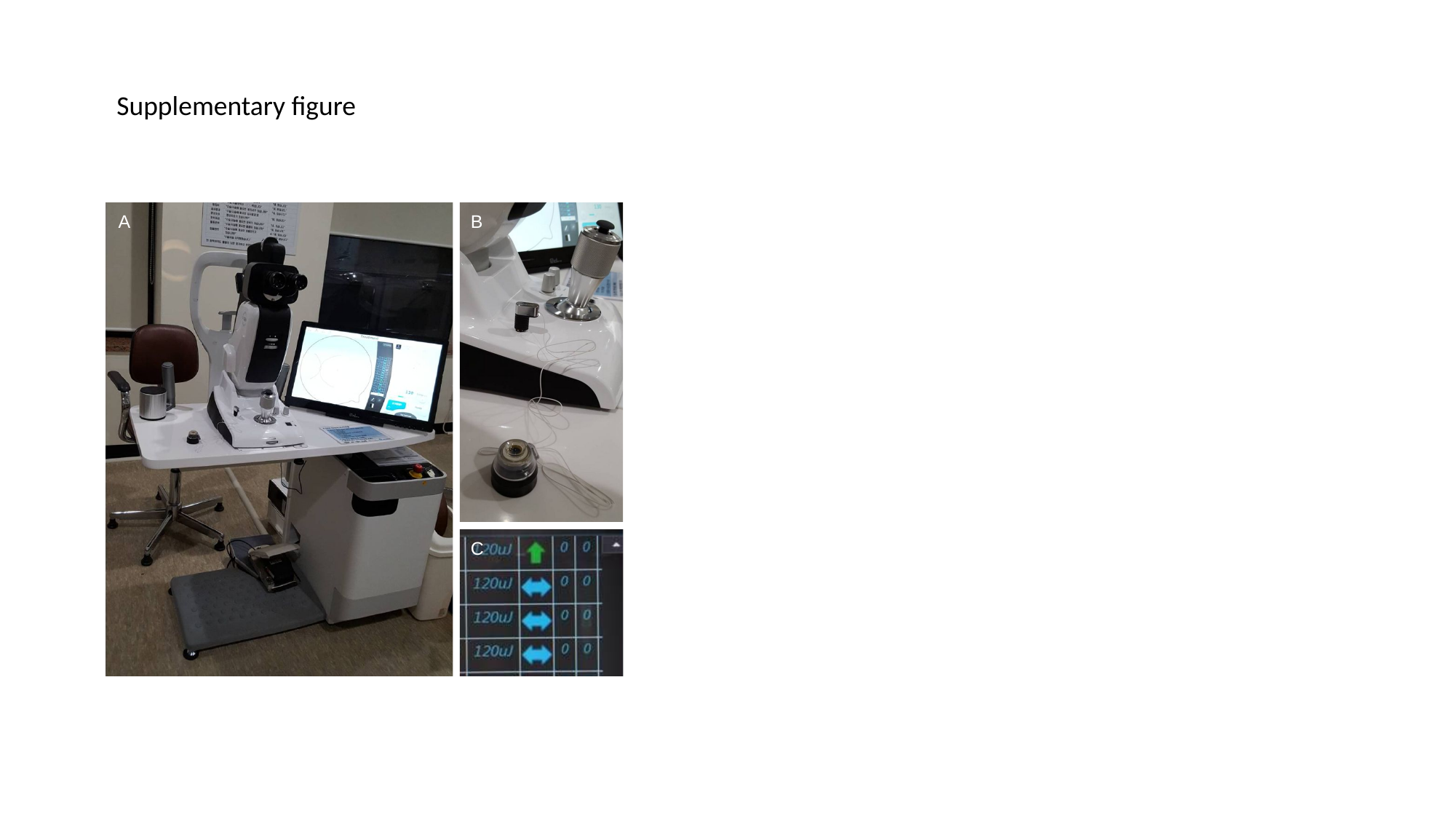

Supplementary figure
A
B
C

Supplement: Supplementary Material — Photographs of the R:GEN selective laser therapy device (Lutronic, Goyang-si, South Korea) used in this study (A). The system includes a combined contact lens/ultrasonic transducer for performing optoacoustic measurements (B). The real-time feedback system control panel displays an up arrow to indicate insufficient laser power. A horizontal double-headed arrow is the okay sign, which indicates microbubble formation (C). [file 6027871.f1.pptx]
